# The role of YWHAZ in cancer: A maze of opportunities and challenges

**DOI:** 10.7150/jca.41316

**Published:** 2020-02-03

**Authors:** Yun Gan, Feng Ye, Xing-Xing He

**Affiliations:** 1 Institute of Liver and Gastrointestinal Diseases, Tongji Hospital, Tongji Medical College, Huazhong University of Science and Technology, Wuhan, China; 2 Department of Pediatrics, Tongji Hospital, Tongji Medical College, Huazhong University of Science and Technology, Wuhan, China

**Keywords:** YWHAZ, cancer, function, molecular mechanism, biomarker

## Abstract

YWHAZ (also named 14-3-3ζ) is a central hub protein for many signal transduction pathways and plays a significant role in tumor progression. Accumulating evidences have demonstrated that *YWHAZ* is frequently up-regulated in multiple types of cancers and acts as an oncogene in a wide range of cell activities including cell growth, cell cycle, apoptosis, migration, and invasion. Moreover, *YWHAZ* was reported to be regulated by microRNAs (miRNAs) or long non-coding RNAs and exerted its malignant functions by targeting downstream molecules like protein kinase, apoptosis proteins, and metastasis-related molecules. Additionally, YWHAZ may be a potential biomarker of diagnosis, prognosis and chemoresistance in several cancers. Targeting *YWHAZ* by siRNA, shRNA or miRNA was reported to have great help in suppressing malignant properties of cancer cells. In this review, we perform literature and bioinformatics analysis to reveal the oncogenic role and molecular mechanism of YWHAZ in cancer, and discuss the potential clinical applications of YWHAZ concerning diagnosis, prognosis, and therapy in malignant tumors.

## Introduction

14-3-3 proteins, which have a molecular mass of around 30 kDa, are a family of highly conserved molecules 1. Seven 14-3-3 isoforms are known to exist—β, γ, ε, η, σ, θ, and ζ—each of which localizes distinctly in tissues with independent isoform-specific functions 1-4. Tyrosine 3 monooxygenase/tryptophan 5-monooxygenase activation protein zeta (also named 14-3-3ζ or YWHAZ), belonging to the 14-3-3 protein family, is a central hub protein involved in many signal transduction pathways and plays a key role in tumor progression 1, 2, 5-8. A growing body of research has demonstrated that YWHAZ was frequently up-regulated and participated in a wide range of cell activities including cell growth, cell cycle, apoptosis, migration/invasion in multiple types of cancers, such as hepatocellular carcinoma, colorectal cancer, lung cancer and breast cancer 5-8. In this review, we seek to summarize the oncogenic role and molecular regulatory network of YWHAZ, with the aim of discovering potential clinical applications of YWHAZ regarding diagnosis, prognosis and treatment in malignant tumors.

## Expression and functions of YWHAZ in cancer

**Figure 1:**
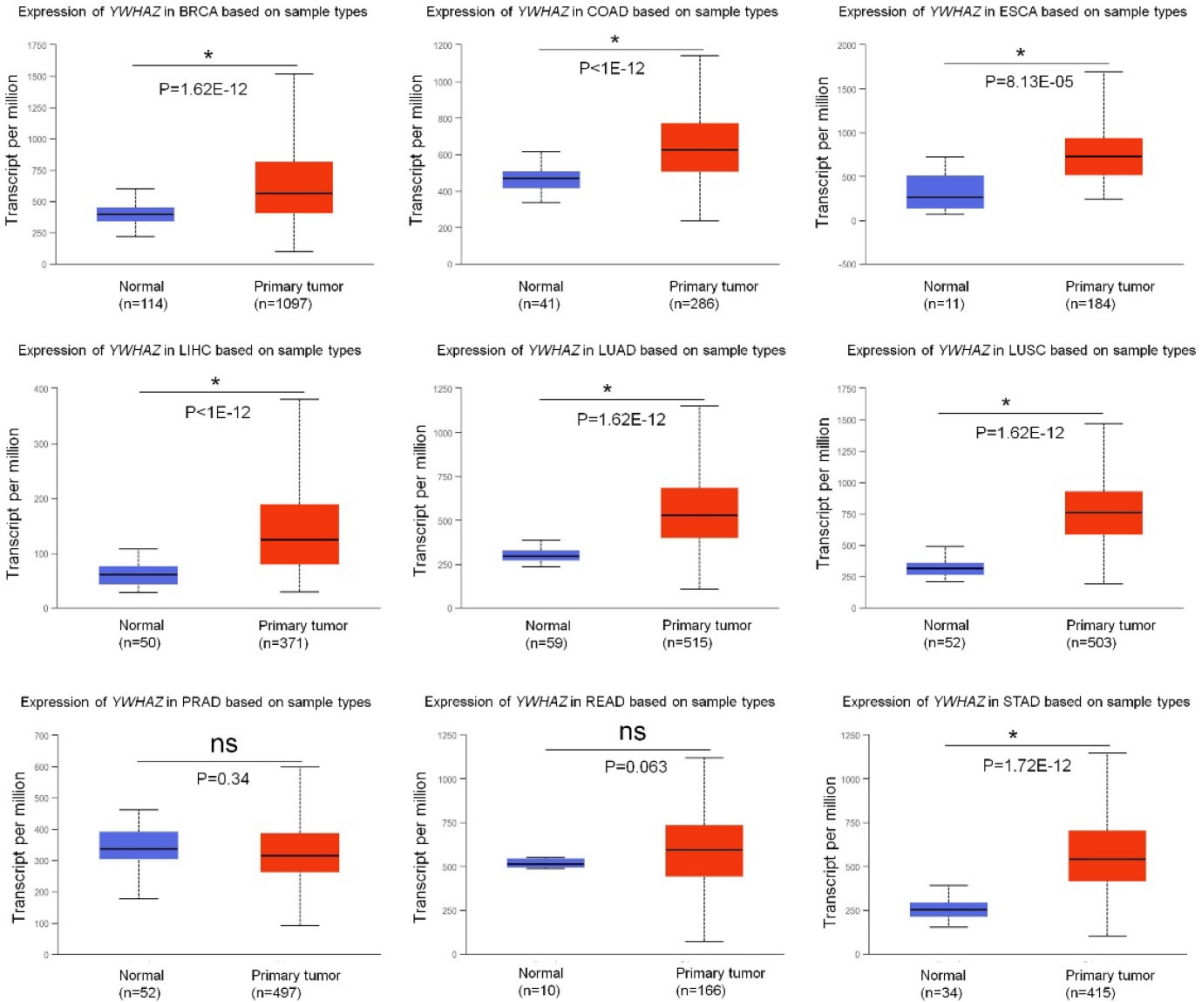
The mRNA level of *YWHAZ* in multiple types of cancers in TCGA samples from UALCAN database. * represents significant difference between two groups. BRCA: breast carcinoma; COAD: colon adenocarcinoma; ESCA: esophagus carcinoma; LIHC: liver hepatocellular carcinoma; LUAD: lung adenocarcinoma; LUSC: lung squamous carcinoma; PRAD: prostate adenocarcinoma; READ: rectum adenocarcinoma; STAD: stomach adenocarcinoma.

Growing researches have reported that *YWHAZ* is frequently up-regulated in multiple types of cancers, acting as an oncogene by promoting malignant properties of cancer cells (summarized in** Table 1**). Using UALCAN database 9, we analyzed the expression of *YWHAZ* in tumor tissues and adjacent tissues from nine types of high-morbidity cancers and observed that *YWHAZ* was significantly increased in breast carcinoma (BRCA), colon adenocarcinoma (COAD), esophagus carcinoma (ESCA), liver hepatocellular carcinoma (LIHC), lung adenocarcinoma (LUAD), lung squamous carcinoma (LUSC) and stomach adenocarcinoma (STAD) (p < 0.0001). However, in prostate adenocarcinoma (PRAD) and rectum adenocarcinoma (READ), there was no significant difference in the expression of *YWHAZ* between cancer tissues and adjacent tissues **(Figure 1)**.

### Hepatocellular carcinoma

We previously examined the mRNA level of *YWHAZ* in 53 pairs of hepatocellular carcinoma (HCC) tissues and adjacent tissues and found that *YWHAZ* was significantly up-regulated in HCC tissues 5. Similarly, *YWHAZ* mRNA expression was higher in eight liver cancer cell lines than normal liver cell line 5. Zhao JF et al. and Chen M et al. likewise verified the high mRNA level of *YWHAZ* in 50 HCC tissues and 374 HCC tissues respectively from The Cancer Genome Atlas (TCGA) database 5, 10. Additionally, YWHAZ protein expression was also higher in 11 of 12 HCC tissues and 8 liver cancer cell lines by western blot and was enhanced in 72 of 135 HCC tissues by immunohistochemical (IHC) 5, 11. Besides, YWHAZ protein level was higher in 10 portal vein tumor thrombus (PVTT) (+) tumors than that in PVTT (-) tumors 12.

Previously, we performed gain- and loss-of-function experiments in liver cancer cells, demonstrating that YWHAZ silencing decreased cell proliferation, clonogenicity, migration/invasion and induced G2 arrest and apoptosis, while YWHAZ up-regulation led to the opposite 5. Choi JE et al. showed that YWHAZ knockdown increased the chemotherapeutic effect of cis-diammined dichloridoplatium through phosphorylation of JNK and p38 11. Lee YK et al. revealed that YWHAZ silencing in liver cancer stem-like cells reduced radio-resistance, leading to decreased cell viability and enhanced apoptosis following γ-irradiation 13. Under both normoxic and hypoxic conditions, down-regulation of YWHAZ reduced invasion capacity, which could be rescued by hypoxia-induced factor-1α (*HIF-1α*) 12. Furthermore, YWHAZ could exert malignant functions by forming complexes with other molecules in HCC 14-16. αB-Crystallin (Cryab) protein, an oncoprotein belonging to the mammalian small heat shock protein family and related with cellular physiology and growth, was up-regulated and formed a complex with YWHAZ, inducing epithelial-mesenchymal transition (EMT) via ERK1/2/Fra-1/slug signaling 14. In Addition, YWHAZ could bind to Axl, promoting Axl-mediated cell migration and invasion 15. However, YWHAZ interference dismissed the mesenchymal phenotype conferred by Cryab overexpression and decreased Gas6/Axl-dependent migration and invasion 14, 15. Song J et al. reported that YWHAZ interacted with heme oxygenase 1 (HO-1) and stabilized HO-1 protein expression by inhibiting its ubiquitin-mediated degradation 16. YWHAZ/HO-1 complex promoted HCC proliferation by signal transducers and activators of transcription 3 (STAT3) signaling pathway 16. Based on the above studies, it may be inferred that YWHAZ overexpression was implicated in HCC progression.

### Colorectal cancer

Li Y et al. observed that the mRNA and protein levels of YWHAZ were both increased in 46 colorectal cancer (CRC) tissues by qRT-PCR and IHC 6. Likewise, YWHAZ protein expression was 1.3-fold higher in COAD stromal tissues than non-cancer stromal tissues by isobaric tags for relative and absolute quantitation-based quantitation proteomics 17. *MiR-451* was down-regulated in colon cancer, and its expression was inversely correlated with YWHAZ, which promoted cell growth through suppression of the nuclear accumulation of FoxO3 6. Additionally, YWHAZ may be responsible for conferring malignant phenotype via extracellular vesicles, while YWHAZ silencing significantly decreased colony formation in CRC cells 18. Thyroid hormone receptor interactor 13 (TRIP13) was reported to interact with YWHAZ and mediate EMT in CRC 19. Knockdown of YWHAZ in TRIP13-overexpressing CRC cells inhibited migration and invasion abilities, as well as decreasing the expression of N-cadherin, β-catenin, snail and increasing the expression of E-cadherin 19.

### Gastric cancer

Guo F et al. reported that the mRNA and protein level of YWHAZ were higher in four gastric cancer (GC) cell lines 20. Enhanced YWHAZ expression was also detected in 6 of 7 GC cell lines (85.7%) by western blot and in 72 of 141 primary GC samples (51%) by IHC 21. In GC, YWHAZ was down-regulated in cells transfected with *miR-375* and luciferase reporter indicated that *miR-375* targets the 3′ UTR of *YWHAZ* 20, 22. Silencing of YWHAZ accelerated *miR-375*-induced apoptosis by caspase-3/ caspase-7 activation and promoted autophagy by PI3K/AKT/mTOR signaling pathway 22, 23, as well as inhibiting cell proliferation, migration/invasion and EMT in GC 20, 21.

### Lung cancer

Deng Y et al. reported that YWHAZ mRNA and protein expression was significantly higher in 152 non-small cell lung cancer (NSCLC) tissues compared to 30 noncancerous lung tissues by qRT‐PCR and IHC 24. Using western blot, Zhao G-Y et al. also detected higher YWHAZ expression in 16 NSCLC tissues than in matched adjacent tissues 25. Chen CH et al. observed that YWHAZ copy number, mRNA and protein expression were all higher in highly invasive lung cancer cell line than less invasive lung cancer cell line 26. Besides, YWHAZ mRNA and protein expression were higher in positive lymph node LUSC patients than that in negative lymph node patients 27.

*In vitro*, proliferation, migration/invasion and EMT were enhanced in lung cancer cells overexpressing YWHAZ 7, 26, 28, while silence of YWHAZ led to the opposite 24, 27, 29. Immunoprecipitation and immunofluorescence analysis revealed that YWHAZ formed complex with Hsp27 protein, colocalizing in the cytoplasm of lung cancer cells 25. Knockdown of this complex suppressed migration of lung cancer cells 25. Additionally, YWHAZ bound with partitioning defective protein 3 (Par3) in lung cancer and loss of Par3 enhanced the interaction of YWHAZ and Tiam1, subsequently activating Rac1 and promoting cancer cell metastasis 30. To confirm YWHAZ function *in vivo*, Chen CH et al. performed three approaches: 1) YWHAZ-cell and control-cell were subcutaneously implanted into the dorsal regions of severe combined immunodeficiency (SCID) mice; 2) YWHAZ-cell and control-cell were injected directly into the circulation of SCID mice to bypass the initial steps of local invasion and intravasation; 3) YWHAZ-cell and control-cell were orthotopically injected into one lobe of SCID mouse lung 26. Tumorigenesis at injection site, local metastasis to the adjunct lobe of the lung, and distant metastasis to the liver were all significantly increased in mice undergone injection of YWHAZ-expressing clone cells 26. Results from these approaches support a role for YWHAZ in promoting cancer metastasis 26. Based on the above studies, malignant transformation of cells induced by increased YWHAZ has been strikingly elucidated in lung cancer.

### Breast cancer

YWHAZ protein expression was assessed by IHC in 139 BRCA tissues and was found to be higher in 45% of BRCA specimens 31. Likewise, Neal CL et al. reported that YWHAZ IHC staining was strongly positive in 42% (n = 51/121) of invasive BRCA specimens 32. TCGA RNA-seq data of 104 corresponding BRCA samples revealed up-regulated *YWHAZ* in cancer tissues compared with adjacent normal tissues 8. Additionally, YWHAZ expression was substantially increased in tamoxifen-resistant BRCA cells compared with chemo-sensitive cells 33.

In BRCA, increased YWHAZ expression had been reported to induce anchorage-independent growth, malignant transformation of cancer cells, and resistance to apoptosis via inhibition of the mitochondrial apoptotic pathway 32. However, Knockdown of YWHAZ greatly decreased cell growth, proliferation, invasion capacity, as well as enhancing tamoxifen-induced inhibition of cell viability and apoptosis promotion 31, 32, 34, 35. Furthermore, YWHAZ can bind to serine 83 on p85, contributing to transformation-related properties of BRCA cells 36. Inhibition of YWHAZ binding to p85 was found to reduce cell proliferation and promote apoptosis 36. In *in vivo* studies, YWHAZ overexpression in FVB mice accelerated the progression of mammary tumors through EMT, angiogenesis promotion and apoptosis inhibition 37. Conversely, delayed tumor onset and reduced tumor growth were observed in mice injected with *YWHAZ* siRNA-treated cells compared with siRNA-control cells 32. To date, combinations of YWHAZ and several oncogenic molecules had been considered to promote transition to invasive breast cancer 38-40. YWHAZ overexpression disrupted the architecture of mammary epithelial cell acini in 3-dimensional culture, resulting in luminal filling, which is a feature of early-stage, benign breast epithelial lesions 38. This progression may be attributed to p53 proteasomal degradation-induced anoikis resistance via the YWHAZ-PI3K-Akt pathway 38. Lu J et al. identified that 8 of the 25 cases (32%) exhibited high levels of both ErbB2 and YWHAZ 39. ErbB2-mediated increase in cell migration and YWHAZ-mediated decrease in cell-cell adhesion via EMT were found to enhance acini invasiveness 39. Co-overexpression of both molecules was considered requisite to induce full transformation, but overexpression of one of these molecules alone was not sufficient to promote progression from ductal carcinoma in situ (CIS) to invasive BRCA and metastasis 39. Unexpectedly, Kambach DM et al. demonstrated that ionizing radiation, oxidative stress and Src-mediated induction of YWHAZ were all capable of inducing invasion of FoxM1-positive cells, even in the absence of ErbB2 expression 40. In summary, these findings strongly support the oncogenic nature of YWHAZ in the promotion of BRCA progression.

### Prostate cancer

In prostate cancer, YWHAZ protein expression was observed to be significantly higher in tumorigenic/metastatic prostate cell lines compared with non-tumorigenic cell line and higher in 50 of 90 prostate cancer tissues than in benign prostate tissues 41, 42. *YWHAZ* mRNA levels showed consistency with protein level 41. It was suggested, through assessment of somatic copy number alterations and IHC, that YWHAZ was noticeably amplified and up-regulated in castration-resistant prostate cancer (CRPC) cases compared with non-CRPC patients 43, 44. Murata T et al. reported that YWHAZ mRNA and protein level was both up-regulated by androgen stimulation 41. Moreover, YWHAZ was associated with the androgen receptor (AR) in the nucleus, promoting AR transcriptional activity 41. Overexpression of YWHAZ promoted cell proliferation and migration in prostate cancer cells, while silencing of YWHAZ showed the opposite 41, 43 . It is well known that YWHAZ dimerization is tightly correlated with its activity in cells, proven to be upstream of rac1 activation 42, 45. Dimerization of increased YWHAZ was found to significantly enhance cell proliferation, viability, and colony formation, while YWHAZ/rac1 complex promoted cell-matrix interactions, lamellipodia formation, cell migration in prostate cancer cell lines 42.

### Other tumors

In acute myeloid leukemia (AML), YWHAZ protein expression was increased in 29 AML patients compared with 24 healthy donors 46. Liang R et al. indicated that YWHAZ mRNA and protein expression was obviously higher in vincristine drug-resistant AML cell line than in AML-sensitive cell line 47. Knockdown of *YWHAZ* by siRNA effectively reduced cell growth and proportion of cells in the S/G2 phases, while increasing the proportion of cells in the G0/G1 phase and enhancing sensitivity to topotecan in both drug-resistant and sensitive AML cells 47. In intrahepatic cholangiocarcinoma (ICC), western blot showed that the protein level of YWHAZ was significantly higher in 30 ICC tissues, and IHC further confirmed the enhanced YWHAZ protein expression in 120 ICC samples 48. Overexpression of YWHAZ was positively related with lymphatic metastasis, tumor-node-metastasis stage, recurrence and the expression of EMT-related markers in ICC 48. Reversely, silence of YWHAZ impaired the invasion, migration, and proliferation of ICC cells 48. In diffuse large B cell lymphoma (DLBCL), 20 of 35 DLBCL cases showed positive expression of YWHAZ and higher YWHAZ was also found in the metastatic T1 DLBCL lymph node tissue compared with the non-metastatic DLBCL tissue and a normal lymph node 49. Moreover, chemotherapeutic mixture consisting of cyclophosphamide, doxorubicin, vincristine, and prednisone (CHOP)-resistant DLBCL cells expressed markedly higher levels of YWHAZ than CHOP-sensitive cells 49. Further study demonstrated that blockade of YWHAZ inhibited DLBCL cell growth, leading to the accumulation of cells in the G2/M phase and restoring the sensitivity of resistant DLBCL to CHOP-induced apoptosis 49.

In summary, *YWHAZ* is frequently up-regulated in various cancers, functioning as an oncogene by promoting the malignant phenotype of cancer cells, particularly through acceleration of migration and invasion.

## Signaling pathways associated with YWHAZ in cancer

### Upstream regulators of YWHAZ

MiRNAs are small, non-coding RNAs of 20-22 nucleotides in length, which are considered to be vital components of gene regulation as important as transcription factors 50. Alterations and dysregulation of miRNAs are often implicated in the initiation and progression of human cancers and are essential for maintaining the malignant phenotype of cancer cells 51, 52. Our team previously proved that *YWHAZ* was a downstream target of *miR-375* and *YWHAZ* expression was negatively correlated with *miR-375* in HCC 5. Ectopic expression of *miR-375* resulted in decreased *YWHAZ*, subsequently accelerating caspase-related apoptosis in gastric carcinoma and repressing telomerase activity in HPV-associated cancers 22, 53. Furthermore, *YWHAZ* expression was enhanced by *miR-451* down-regulation, subsequently regulating a series of cell activities, including cell proliferation, survival, apoptosis and endocrine chemoresistance 6, 8, 33, 46, 54. For example, low *miR-451*/high *YWHAZ* expression was observed to promote cell proliferation and inhibit apoptosis through AKT targeting in AML and activate growth factor receptors and kinases (HER2, EGFR, AKT, and MAPK) involved in endocrine resistance in breast cancer 46, 54. Conversely, negative regulation of *YWHAZ* via high *miR-451* expression greatly reduced cell proliferation and growth and induced cell-cycle arrest alongside apoptotic cascade in breast cancer 8, 54. Li Y et al. elucidated that decreased *YWHAZ* expression via *miR-451* activity inhibited cell growth in colorectal cancer through nuclear accumulation of FoxO3 6. FoxO3 had been verified as a key protein in the suppression of cancer progression, with roles including control of differentiation and tumorigenicity through the PI3K/Akt/mTOR and MEK/ERK signaling pathways 55-57. Nuclear accumulation of FOXO3a could be promoted by *miR-22* and was observed to subsequently reverse invasive phenotype of HCC cells through repression of YWHAZ-mediated AKT phosphorylation 10. Besides, the expression of *YWHAZ* could be negatively regulated by *miR-30c* in cervical cancer, by *miR-544* in breast cancer, and by *miR-613* in HCC 58-60.

Recently, long non-coding RNAs, more than 200nt and involved in multiple cell processes, are emerging as competing endogenous RNAs to regulate *YWHAZ* by targeting miRNAs 61-63. In gastric cancer, long non-coding RNA *LUCAT1* was negatively correlated with *miR-134-5p* and *miR-134-5p* was negatively related with *YWHAZ* 61. Knockdown of *LUCAT1* inhibited *YWHAZ* expression, which can be reversed by *miR-134-5p* inhibitor 61. Similarly, long non-coding RNA *SNHG14*, acting as a *miR-206* sponge and decreasing its expression, increased *YWHAZ* expression in cervical cancer 62 and long non-coding RNA *LINC00858* regulated *YWHAZ* by inhibiting *miR-22-3p* in colorectal cancer 63.

### Downstream targets of YWHAZ

#### YWHAZ and protein phosphorylation

The 14-3-3 family interacted with a diverse range of cell signaling proteins by binding to an amphipathic helix and activating it through phosphorylation 64, 65. Doubly phosphorylated peptides tightly bound simultaneously at adjacent 14-3-3 sites to form high-affinity bidentate complexes 65. YWHAZ was found to play an important role in chemoresistance through modulation of protein phosphorylation. In HCC, αB-Crystallin-YWHAZ complexes were observed to promote EMT through elevated ERK1/2 phosphorylation, which impaired the effect of sorafenib, while JNK and p38/MAPK phosphorylation were verified to increase chemosensitivity of HCC cells to CDDP when YWHAZ was silenced 11, 14. Moreover, Cdc2, belonging to the cyclin-dependent kinase family, is a maturation-promoting factor involving in the G2-M transition 66. Cdc2 phosphorylation had been observed after YWHAZ reduction, subsequently sensitizing lung cancer cells to cisplatin-induced G2-M arrest 67.

#### YWHAZ and apoptosis protein

Pro-apoptotic proteins Caspase-3 and Bax were increased in YWHAZ-depleted liver cancer stem-like cells (CSCs) 13. Conversely, a dramatic loss of Bax and caspase-3 were observed in breast cancer cells overexpressing YWHAZ 38. Neal CL et al. also found that decreased YWHAZ sensitized breast cancer cells to apoptosis in low serum conditions by increasing cytochrome C release, subsequently reducing procaspase 9 expression and caspase substrate cleavage 32. These studies demonstrated that YWHAZ may induce apoptosis resistance by modulating mitochondrial apoptosis pathways.

#### YWHAZ and metastasis-related molecules

ErbB2, a receptor tyrosine-protein kinase, was overexpressed in approximately 20%-30% of BRCA and played a vital role in the development and metastasis of BRCA 68, 69. Co-overexpression of YWHAZ and ErbB2 in ductal CIS conferred an increased risk of progression to invasive BRCA than those overexpressed one molecule alone 39. This was believed to occur through activation of the TGF-b/Smads pathway, which subsequently led to ZFHX1B/SIP-1 up-regulation, E-cadherin loss, and EMT 39. Kambach DM et al. also demonstrated that ionizing radiation-induced YWHAZ upregulation was required and sufficient for cell invasion in ErbB2-positive BRCA cells, together with FoxM1 40. Transforming growth factor-β (*TGF-β*) functions as a tumor suppressor in premalignant cells but, interestingly, as a metastasis promoter in cancer cells 70. In breast cancer cells, *YWHAZ* destabilized *p53* and stabilizes *Gli2*, promoting *TGF-β*-induced bone metastasis 70. Binding of Axl to YWHAZ caused Smad3L phosphorylation and then resulted in the up-regulation of *TGF-β* target genes and *TGF-β1* in mesenchymal HCC cells, which is essentially required for Axl-mediated cell invasion 15. Under hypoxia condition, HIF-1α could be induced, acting as a crucial factor for tumor metastasis in HCC 71. YWHAZ enhanced HIF-1α protein stability and recruited HDCA4 to inhibit HIF-1α acetylation, subsequently promoting HCC cell metastasis via HIF-1α/EMT or PI3K/Akt/NF-кB signaling pathway 12, 71.

**Figure 2:**
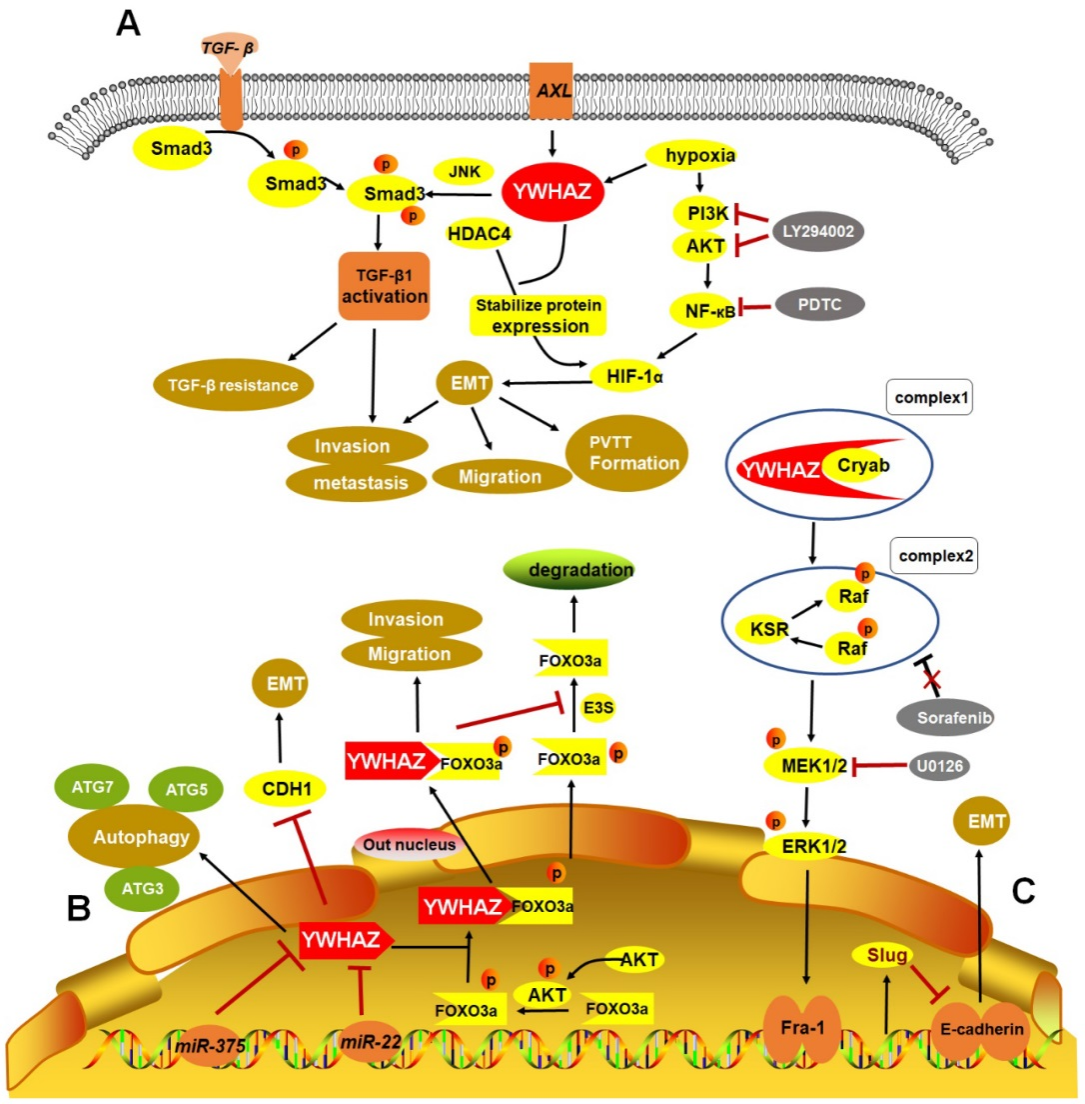
Verified signaling pathways of YWHAZ in HCC. **A.** Phosphorylation of Smad3 linker region by Axl/YWHAZ and JNK activates the expression of *TGF-β1*, leading to HCC invasion and metastasis by *TGF-β* resistance. Additionally, under hypoxic circumstance, YWHAZ interacts with HIF-1α and enhances HIF-1α protein stability by recruiting HDAC4 and activating PI3K/Akt/NF-кB pathway, then inducing cell migration, invasion and PVTT formation in HCC. **B.** *MiR-375*, *miR-22* directly targets 3′-UTR of *YWHAZ* mRNA to inhibit *YWHAZ* expression. YWHAZ can induce autophagy by ATG3, ATG5, ATG7 and promote EMT by suppressing CDH1 in HCC. Additionally, phosphorylated AKT inhibits the activity of FOXO3a by promoting its phosphorylation and binding YWHAZ with FOXO3a, then promoting HCC migration and invasion. In cytoplasm, YWHAZ/FOXO3a complex inhibits the dephosphorylation of phosphorylated FOXO3a and promotes FOXO3a degradation. **C.** Cryab complexes with YWHAZ and elevates its expression, leading to activation of ERK1/2/Fra-1/slug signaling pathway and then inducing EMT progression by decreasing E-cadherin expression. Moreover, sorafenib response is impaired in this signaling pathway. HCC: hepatocellular carcinoma; TGF-β: transforming growth factor-β; HIF-1α: hypoxia-induced factor-1α; EMT: epithelial-mesenchymal transition; PVTT: portal vein tumor thrombus; Cryab: αB-Crystallin.

Taken together, some crucial upstream regulators and downstream targets of YWHAZ involving in cancer progression were summarized in **Table 1**. Importantly, three HCC RNA-seq datasets (GSE69164, GSE63863, and GSE55758) from Gene Expression Omnibus (GEO) indicated that *YWHAZ* is a hub gene in HCC 72. Hence, we summarized the verified signaling networks of YWHAZ in HCC to systematically understand its role 5, 10, 12, 14, 15 **(Figure 2)**.

## YWHAZ as a potential biomarker in cancer

### Diagnosis

In the past decades, the diagnostic potential of YWHAZ had aroused considerable interest. Liu M et al. detected that the prevalence of YWHAZ autoantibodies was 16.7% (28/168) in HCC, significantly higher than in liver cirrhosis, chronic hepatitis, and normal human sera by enzyme-linked immunosorbent assay (ELISA) analysis (P<0.01) 73. Similarly, ELISA showed that autoantibody to YWHAZ was obviously higher in 465 gastric cancer patients (0.17 ± 0.08 ng/ml) compared to 465 normal samples (0.14 ± 0.06) (P<0.001) 74. Moreover, YWHAZ autoantibody combined with diagnosis biomarkers of gastric cancer (CEA, CA199, CA724), increasing the diagnostic sensitivity to 52.7% 74. Zhang Y et al. discovered that *YWHAZ*, as well as *HTR2B*, *CHL1*, the *ZNF* family and *FYN*, were observed to be most obviously altered between 46 liver metastatic uveal melanoma samples and 45 non-metastatic uveal melanoma samples, derived from GEO database 75. This distinction may provide diagnostic and preventative worth for uveal melanoma liver metastases in the future 75. Huang Y-D et al. identified genes related to bladder cancer using microarray chip, detecting that *YWHAZ*, *PRDX2* and *C1QBP* were all related to inflammation and cell proliferation and could be regarded as candidate biomarkers for bladder cancer diagnosis 76. In conclusion, these studies demonstrated that YWHAZ or combination of YWHAZ and clinical markers may be promising diagnostic biomarker in the future.

### Prognosis

**Figure 3:**
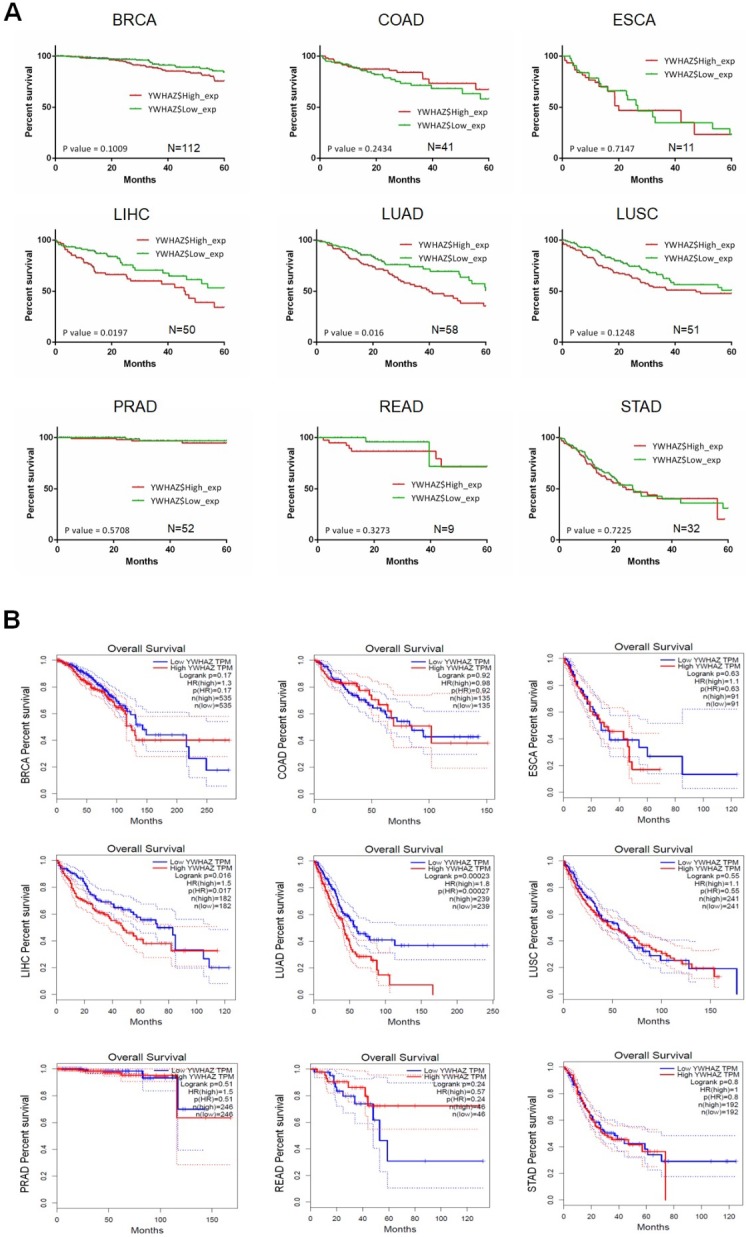
The correlation between *YWHAZ* expression and overall survival in multiple types of cancers. The x axis is the overall survival month, and the y axis represents the survival rate. **A.** Kaplan-Meier survival analysis of *YWHAZ* at 60 months is shown. These data were derived from TCGA database. Group cutoff is quartile. **B.** Overall survival analysis of *YWHAZ* from GEPIA database. Group cutoff is median. Abbreviations are as marked in Figure 1.

**Figure 4:**
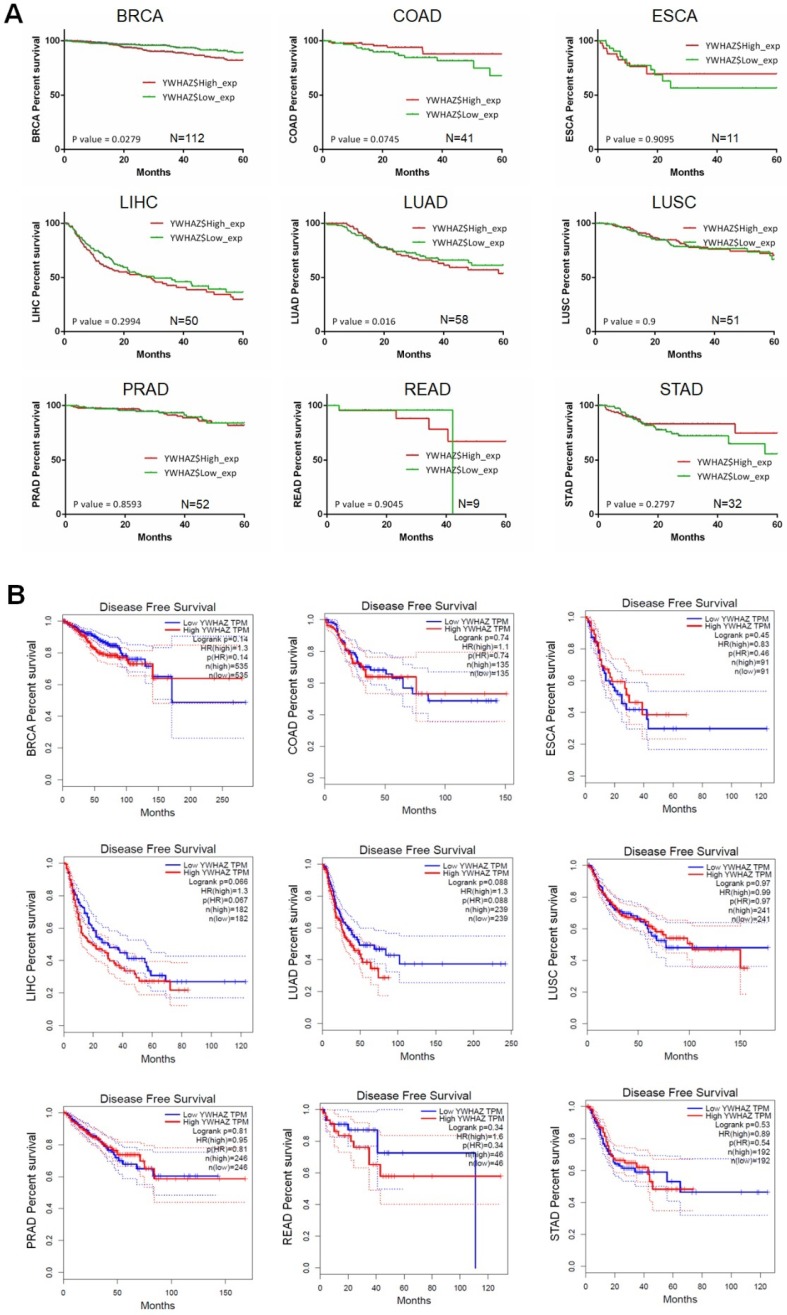
The correlation between *YWHAZ* expression and disease-free survival in multiple types of cancer. The x axis is the disease-free survival month, and the y axis represents the survival rate. **A.** Kaplan-Meier survival analysis of *YWHAZ* at 60 months is shown. These data were derived from TCGA. Group cutoff is median. **B.** Disease-free survival analysis of *YWHAZ* from GEPIA database. Group cutoff is median. Abbreviations are as marked in Figure 1.

Our team used Kaplan-Meier survival analysis to explore the relationship between *YWHAZ* expression and overall survival/disease-free survival at 60 months in BRCA, COAD, ESCA, LIHC, LUAD, LUSC, PRAD, READ, and STAD from the Cancer Genome Atlas (TCGA) database. As can be seen in **Figure 3A and Figure 4A,** *YWHAZ* expression was significantly correlated with overall survival at 60 months in LIHC (p = 0.0197) and LUAD (p = 0.016), and with disease-free survival at 60 months in BRCA (p = 0.0279) and LUAD (p = 0.016). We also conducted Kaplan-Meier survival analysis in Gene Expression Profiling Interactive Analysis (GEPIA) database with larger samples 77, determining that overall survival time was remarkably longer in LIHC (p = 0.016) and LUAD (p = 0.00023) with low *YWHAZ* expression (**Figure 3B**) 24, which was consistent with the result of TCGA. However, there was no statistic difference of disease-free survival between high *YWHAZ* and low *YWHAZ* samples (**Figure 4B**).

In HCC, we observed that combination of *ASH1*, *miR-375* and *YWHAZ* resulted in significant differences regarding overall survival at 50 months (p = 0.003), 60 months (p = 0.0096) and 100 months (p = 0.0158) 5. Yufu T et al. demonstrated differences (P < 0.001) in both overall survival (28 vs. > 33 months) and time to recurrence (12 vs. 24 months) between high HIF-1α/YWHAZ vs. low HIF-1α/ YWHAZ HCC groups 12. Furthermore, decreased survival (P = 0.025) was also considered to be strongly associated with elevated levels of YWHAZ and Axl in HCC 15. Fan T et al. reported that overall survival at 5 years after surgery and cancer-specific survival in stage I NSCLC YWHAZ-positive patients were 0.36 and 0.60, compared with 0.68 and 0.95 in YWHAZ-negative patients 67. LUSC patients with high YWHAZ/ TGFβ receptor types 1 (TGFβR1) have shorter overall survival than patients with low YWHAZ/TGFβR1 27. Similarly, YWHAZ overexpression was significantly associated with reduced disease-free survival/overall survival and earlier time to disease recurrence, and death in breast cancer by combining with elevated levels of Akt, FOXM1, ErbB2, LOC441453 and LAPTM4B 31, 32, 35, 36, 39, 78-80. In particular, YWHAZ overexpression, ErbB2 overexpression, and positive lymph node status were seen to be independent prognostic factors in breast cancer 39. In head-and-neck/oral squamous cell carcinoma, disease-free survival of the YWHAZ-positive group was 23 months compared with 35 months for the YWHAZ-negative group 81. In glioblastoma, 2-year overall survival and median survival time in the YWHAZ-positive group were 8.6% and 12.9 months, compared with 16.7% and 17.9 months in the YWHAZ-negative group 82. Furthermore, a growing number of studies have proposed that elevated YWHAZ expression was correlated with poor prognosis in prostate cancer 44, ICC 48, and gastric carcinoma 21, implying that YWHAZ was tightly associated with the survival of cancer patients.

### Chemoresistance

It is well known that barriers to chemotherapeutic agents during cancer therapy include intrinsic and acquired resistance, thus the effect of chemotherapy among cancer patients is still often sub-optimal. The anti-apoptosis ability exerted by YWHAZ may be responsible for chemoresistance. High levels of YWHAZ had been found in CHOP-resistant DLBCL cells and 9-nitrocamptothecin resistant prostate cancer cells, compared with chemo-sensitive cells 49, 83. Intriguingly, YWHAZ knockdown had been shown to restore the sensitivity of resistant cells to apoptosis induced by chemotherapeutic agents including CHOP, 9-nitrocamptothecin, CDDP, cisplatin and TPT 11, 47, 49, 67, 83. In breast cancer, silencing of *LAPTM4B* and *YWHAZ* gene sensitized tumor cells to anthracyclines, while overexpression of these genes induced drug resistance 79. Moreover, YWHAZ knockdown enhanced the growth inhibitory effects of SERMs in endocrine-resistant breast cancer cells, restoring sensitivity to endocrine treatments 35, 54. Based on the above evidence, it is promising to target YWHAZ to decrease chemoresistance and improve the effect of chemotherapy.

## Therapeutic potential

Surgery, chemotherapy and radiotherapy have traditionally been the main therapeutic methods for human cancers. However, the prognosis of most cancer patients treated through these approaches still remain fairly poor. Given the oncogene role of YWHAZ in multiple cancers, the combination of traditional therapeutic methods and *YWHAZ*-targeted therapies may be an attractive project in the future.

Our team delivered si-NC, si-*YWHAZ* and si-*YWHAZ*/DOX using nanoliposomes (L) in established mouse HCC xenograft models, observing that tumor growth could be inhibited in the latter two groups compared with control 5. IHC analysis further revealed that cell proliferation was inhibited and cell apoptosis was increased *in vivo* by YWHAZ blockade 5. Neal CL et al. observed delayed breast cancer onset and reduced tumor growth in mice injected with *YWHAZ* siRNA using lipofectamine 32. Similarly, nude mice were inoculated with lung cancer cells, shRNA-control lung cancer cells and sh-*YWHAZ* lung cancer cells using lipofectamine 67. Results of the three groups showed that tumor volumes were 169.49 ± 20.61, 154.54 ± 20.06, and 151.49 ± 34.78 mm³ after 17 days (P = 0.091) and tumor growth ratios were 54%, 50% and 22% by 28 days after the initiation of cisplatin treatment, implying a suppressive role of YWHAZ knockdown 67. Yufu T et al. used HCC-CSQT-2/sh-*YWHAZ* cells, which are derived from PVTT and prone to form PVTT, to establish orthotopic transplantation assays in nude mice 12. Using these techniques, they established mouse models of PVTT by injecting HCC-CSQT-2/sh-*YWHAZ* cells or HCC- CSQT-2/sh-control cells into mice through the tail vein 12. Results from this study indicated that blockade of *YWHAZ* by shRNA suppressed lung metastases and formation of PVTT *in vivo* 12. Therapeutic approaches which increase expression of microRNAs targeting *YWHAZ* might also be worth exploring. Up-regulation of *miRNA-451* by murine stem cell virus vector directly decreased *YWHAZ* expression and inhibited colon cancer growth* in vitro* and *in vivo* 6. However, compensatory effects of siRNA, shRNA or miRNA approach by targeting a single molecule do exist after long term treatment. Recently, proteolysis targeting chimera (PROTAC) technology has attracted people's interest for its promise in disease therapeutics that induced targeted protein degradation and has made success in a selective small-molecule degrader of STAT3 which achieved complete tumor regression *in vivo* 84. Thus, chemical compound or molecular inhibitors targeting *YWHAZ* specifically are greatly needed in the future.

## Conclusion and future direction

To date, *YWHAZ* has been shown to be frequently up-regulated and function as an oncogene by regulating multiple signaling pathways in cancers (**Table 1**). YWHAZ overexpression is regulated by miRNAs or long non-coding RNAs and activates downstream molecules, including protein kinases, apoptosis proteins, and metastasis-related molecules, to facilitate the malignant potential of cancer cells. However, a comprehensive assessment of YWHAZ regulatory networks through bioinformatics analysis is warranted. Growing evidences suggested the potential role of YWHAZ in cancer diagnosis, prognosis and chemoresistance. However the specificity and sensibility of YWHAZ as an independent biomarker are limited. Combinations of YWHAZ with other cancer-specific molecules may have better ability to serve as biomarkers. At present, *YWHAZ* targeting therapy alone through siRNA, shRNA or miRNA to delay tumor development shows some preliminary results. Nevertheless, safer and more effective carriers for YWHAZ inhibitor delivery, or combinations of YWHAZ with other promising therapeutic targets are greatly needed. In summary, *YWHAZ*, acting as an important oncogene, is increasingly showing its potential as a biomarker for diagnosis, prognosis, chemoresistance and therapeutic target in a diverse range of malignancies.

## Figures and Tables

**Table 1:** Functions and relevant molecular mechanisms of YWHAZ

Cancer type	Function of YWHAZ	YWHAZ complex	Upstream regulators of YWHAZ	Downstream targets of YWHAZ	Reference
Hepatocellular carcinoma	Enhanced cell proliferation, colony formation, migration/invasion, EMT, chemoresistance; Inhibited cell apoptosis	Bound with αB-Crystallin; Axl; HO-1	*miR-22; miR-375; miR-451a; miR-613*	AKT; ERK1/2; Caspase-3; Bax; Smad3; *TGF-β*; HDCA4; *HIF-1α*; JNK and P38; STAT3; ATG7;*P53*;E-cadherin	5, 10-16, 60, 71, 85
Colorectal cancer	Promoted cell growth, colony formation, migration, invasion, EMT	Interacted with TRIP13	*miR-451; LINC00858; miR-22-3p*	FoxO3; N-cadherin; β-catenin; snail; E-cadherin	6, 18, 19, 63
Gastric carcinoma	Promoted cell proliferation, migration/invasion, EMT; Inhibited cell apoptosis	/	*miR-375; LUCAT1; miR-134-5p*	PDK1/Akt; Caspase-3; Caspase-7; wnt/β-catenin; E-cadherin; N-cadherin; Vimentin; PI3K/AKT/mTOR	20-23, 61
Lung cancer	Promoted cell proliferation, EMT, migration/invasion; Inhibited cell apoptosis	Bound with heat shock protein27; β-catenin; Par3; Tiam1	/	β-catenin; Protein kinase C/NF-κB and Snail; E-cadherin; N-cadherin; Vimentin; TGFβR1; MUC1	7, 24-30
Breast cancer	Induced cell proliferation, colony formation, metastasis /invasion, chemoresistance; Inhibited cell apoptosis	Bound with ErbB2; p85	*miR-193b; miR-451; miR-30c*	Caspase-3;Bax; PI3K/Akt; TGF-b/ Smad; ZFHX1B; TbRI; FOXM1; HER2; EGFR; MAPK; *miR-221*; c-Jun; β-catenin	8, 31, 32, 34-40, 54, 58
Prostate cancer	Promoted cell proliferation, colony formation, migration/invasion; Inhibited cell apoptosis	/	/	Rac1	41-44
